# Study on sand liquefaction induced by Songyuan earthquake with a magnitude of M5.7 in China

**DOI:** 10.1038/s41598-022-13549-8

**Published:** 2022-06-10

**Authors:** Ping Li, Zhaoyang Tian, Jingshan Bo, Sheng Zhu, Yuying Li

**Affiliations:** 1 Key Laboratory of Earthquake Engineering and Engineering Vibration, Institute of Engineering Mechanics, China Earthquake Administration, Haerbin, 150081 Heilongjiang China; 2 Institute of Engineering Mechanics, China Earthquake Administration, Haerbin, 150081 Heilongjiang China; 3 Institute of Disaster Prevention, Sanhe, 065201 Hebei Province China; 4 Hebei Key Laboratory of Earthquake Disaster Prevention and Risk Assessment, Sanhe, 065201 Hebei China

**Keywords:** Natural hazards, Seismology

## Abstract

A large-scale sand liquefaction producing typical and novel surface phenomena was found at the epicenter of Songyuan M5.7 earthquake occurring on May 28, 2018. Field survey and experimental test encompassing boring sampling, standard penetration test (SPT), cone penetration test (CPT), scanning electron microscopy (SEM), X-ray diffraction (XRD), and X-ray fluorescence (XRF) were performed to ascertain the liquefaction damage and site characteristic. Cone penetration test is an excellent assay for the identification of liquefied sand layer and acquisition of physio-mechanical parameter. Moreover, the assay is applicable for in-situ post-earthquake investigation. Factors promoting the formation and controlling the distribution of the sand liquefaction were analyzed. The liquefaction impacted an 80 km2 area, and was primarily embodied as sand boil and water sprout on rice field, despite producing no significant structural damage. Due to the simple profile of local soil layer, ground motion, geomorphic condition, and groundwater level were the main factors governing the distribution of the liquefaction. Majority of the liquefied sand layer was discovered at the depth less than 10 m. However, deep layer liquefaction at the depth greater than 18 m was also discovered, which was demonstrated by the upward movement of liquefied sand towards the upper silty clay layer at the depth of 17 m. Most importantly, we have identified loess liquefaction, a phenomenon which had not been reported previously in Northeast China. Lastly, it is important to highlight the risk of significant liquefaction damage at Songyuan. Hence, investigating the liquefaction risk is potentially beneficial for augmenting planning on earthquake mitigation, engineering reconnaissance, and design project.

## Introduction

Liquefaction is a highly-destructive geological disaster induced by earthquake. History has recorded several liquefaction damage promoted by earthquake, such as the liquefaction damage in Niigata (Japan, 1964), Dagupan City (the Philippines, 1990), Chi-Chi (Taiwan, 1999), Kocaeli (Turkey, 1999), and Wenchuan (China, 2008). The destructive impact of liquefaction such as landslide promotes the collapse of buildings and thus the failure to achieve the targeted structural service life. Thus, ongoing study has been expanding our understanding on liquefaction, encompassing liquefaction mechanism, evaluation, and mitigation^1–6^.

Post-earthquake investigation is an essential research methodology to study the distribution of liquefaction and identify the influencing factors of a liquefaction. There are two main objectives of the investigation: Initially, liquefied and non-liquefied region are determined based on ground damage. Successively, ground motion data are collected and quantitative data for detailed calculation and analysis are obtained by conducting drilling, screening test, standard penetration test, cone penetration test, and wave velocity test. Researchers and earthquake engineers deduce detailed information on the damage due to liquefaction and its related seismic activity by investigating liquefaction macrophenomena.Nevertheless, there are several limitations to previous investigation on liquefied macrophenomena: (1) While liquefied sites were identified during preliminary post-earthquake investigation by the observed surface macrophenomena such as sand boil, deep soil liquefaction with no obvious macrophenomena was frequently neglected. (2) Report on in-situ special soil liquefaction in Northeast China, such as liquefaction of loess, is scarce. An earthquake with a magnitude of M5.7, the moment magnitude is M_W_ 5.2, occurred at Songyuan City, Jilin Province, China on 28th May, 2018. The earthquake was felt throughout Jilin Province, part of Heilongjiang Province, and Inner mongolia autonomous region. The epicenter of the earthquake was the Yamutu village, which is located at the alluvial terrace of Songhua River. The sub-surface soil layer is mainly composed of fine sand and the water level is shallow. Thus, the occurrence of earthquake-induced liquefaction is increasing in recent years. In order to investigate the liquefaction distribution and damage, field survey and subsequent experimental test were conducted, involving boring sampling, standard penetration test (SPT), cone penetration test (CPT), scanning electron microscopy (SEM), X-ray diffraction (XRD), and X-ray Fluorescence (XRF). This paper initially provides an overview on the seismic characteristic of Songyuan and preliminary data on the characteristic of M5.7 earthquake. The earthquake history and current ground motion at Songyuan are elaborated. Thereafter, observed phenomena at liquefied and non-liquefied sites are presented. Not only typical liquefaction damages such as sand boil and waterspout were found, interesting novel phenomena such as deep soil liquefaction and special soil liquefaction (i.e. loess liquefaction) were also indicated. Hence, the outcome of experimental series performed to ascertain the novel finding in Northeast China is discussed. Overall, this paper provides a better understanding on the prevention of liquefaction at Songyuan and fundamental understanding for future research.

## Seismic characteristic of Songyuan

### Historical record of earthquake

**Figure 1:**
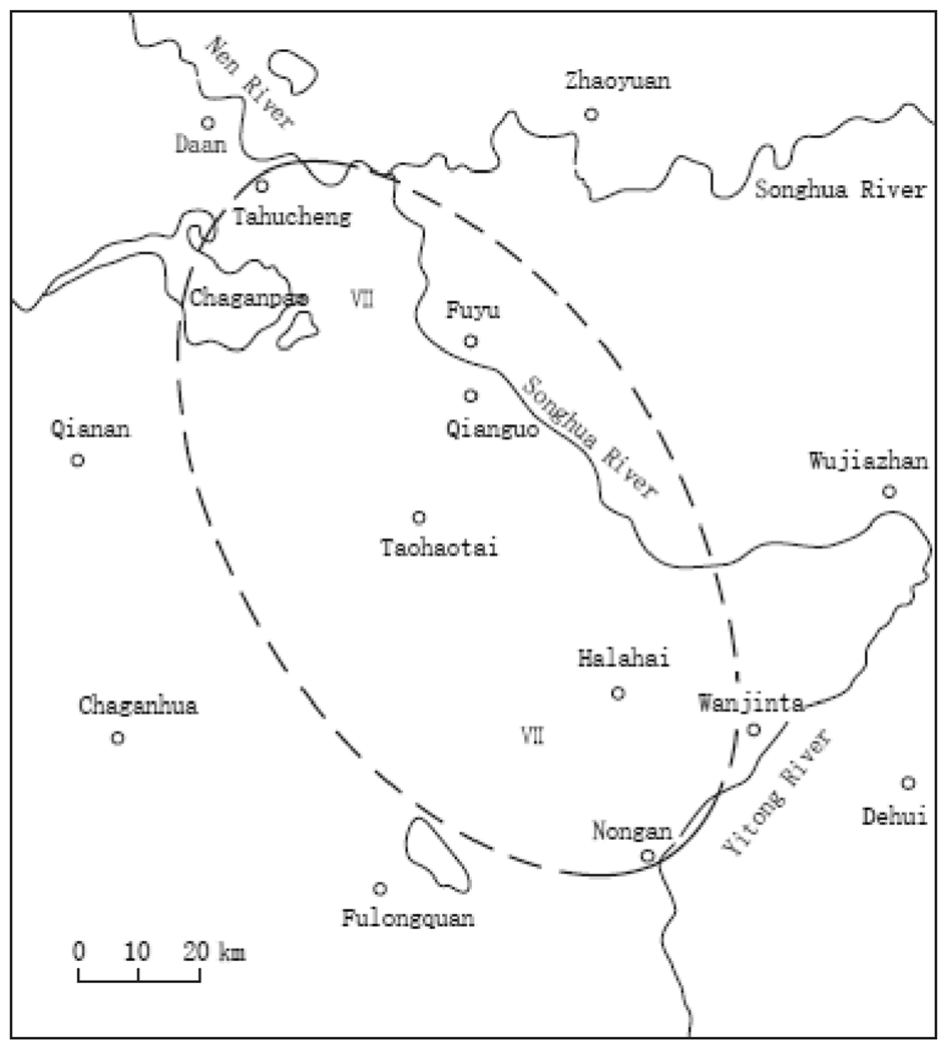
Isoseismal map of Qianguo earthquake occurring on 1119 at the surrounding region of Songyuan city. Adapted from Wu et al. We created this figure in using ArcGIS 10.0 (URL: http://www.esri.com/).

Songyuan is a city located at the Southern Songliao Basin. It has a complex fault structure and is one of the two cities in Northeast China which is fortified against seismic intensity of VIII. Series of earthquake with the magnitude of M > 5.0 have occurred throughout the city’s history. As mentioned in *Dajin chorography*, a destructive earthquake (Qianguo earthquake) with an estimated magnitude of (insert magnitude here) occurred on February 1119, and is documented as the most devastating earthquake attacking the city. Figure 1 illustrates the isoseismal map of the earthquake^7^. The epicentral intensity was estimated between VIII and IX. Thousands of people died of the generated subsidence, accounting for approximately one-tenth of the combined population of Zhaozhou and Longzhou. The earthquake impacted the surrounding region of Songhua River Basin where Holocene sand layer is abundantly distributed. The shallow sand layer is saturated and loose, thus being susceptible to liquefaction under an earthquake. Despite the regional geomorphic feature, there was no large-scale landslide, ground crack, or other subsidence-related phenomena in the area. It is suggested that the large-scale subsidence was induced by sand liquefaction spanning from Tahu Town to Nongan Town.

In recent years, seismic activity with a magnitude of M4.0–5.0, particularly multiple earthquake swarm, frequently occurs around Songyuan^8,9^. Several earthquake have happened since 2006, comprising of: (1) Qianan earthquake with a magnitude of M5.0 on 31st March 2006, (2) Qianguo earthquake swarm with a magnitude of M > 5.0 on 2013, (3) Ningjiang earthquake with a magnitude of M4.9 on 23rd July 2017, and (4) Songyuan earthquake with a magnitude of M5.7 on 28th May 2018. It is important to emphasize that the Qianguo earthquake swarm occurred repeatedly with varying intensity, beginning with a magnitude of M > 5.0 on 31st October 2013 and ended with a magnitude of M5.5 at the end of 2014. During the time period, five earthquake with the magnitude of M5.0–5.9 and seven earthquake with the magnitude ofM4.0–4.9 hit the region. The multiple earthquake affected tens of thousands, and caused damage to buildings and farmland. Thus, it is necessary to highlight the severe liquefaction risk at the region around Songyuan, noting the widely distributed shallow sand layer and underground water.

### Preliminary data on the characteristic of M5.7 earthquake

**Figure 2:**
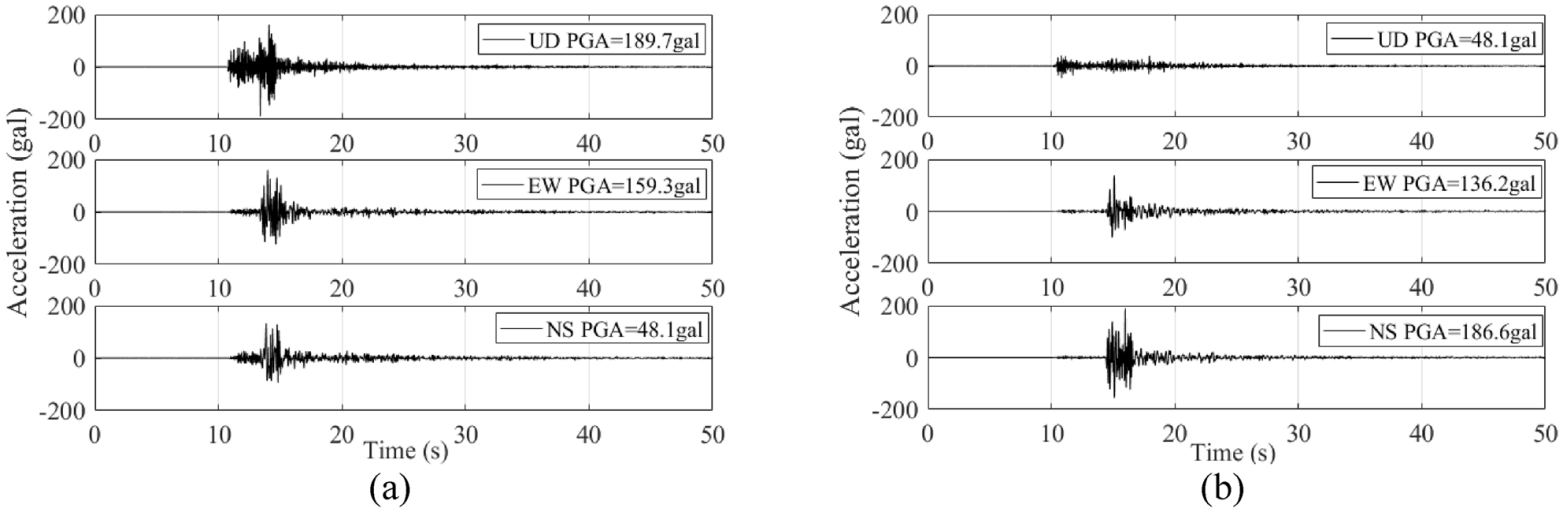
Strong motion record documented at (**a**) Daliba and (**b**) Fenghua station.

Focal mechanism solution (FMS) is an excellent tool to analyze the mainshock-aftershock wave forms recorded as seismographs during an earthquake. Focal mechanism solution of seismographs generated during the Songyuan M5.7 earthquake indicated that it was a strike-slip earthquake. It was suggested that Fuyu-Zhaodong fault was the seismogenic fault. Figure 2 is the seismic intensity distribution map from seismic intensity survey. The highest intensity was VII, covering a 157 km^2^ area. The long axis of the region experiencing the intensity of VII was distributed along the northeast direction as long as 9.2 km, whilst the short axis was 4.8 km-long. On the other hand, the region experiencing an intensity of VI spanned an 880 km^2^ area. The long axis was 24 km-long and the short axis was 16 km-long. The intensity distribution was north–south asymmetry, and the decay rate of intensity was greater towards the southwest than the northeast.

The FMS analysis was supplemented by strong motion record. Figure 2 illustrates the strong motion record with peak ground acceleration (PGA) over 100 cm/s^2^, documented at Daliba and Fenghua station by China Strong Motion Network Centre. The two stations were located at close proximity to the epicenter. Daliba station is located 16 km away from the epicenter, and recorded horizontal and vertical PGA of 159 and 189 cm/s^2^ respectively. On the other hand, Fenghua station is located 24 km away from the epicenter, and recorded horizontal and vertical PGA of 189 and 48 cm/s^2^.

## Field investigation and liquefaction macro phenomenon

### Distribution of liquefaction

**Figure 3:**
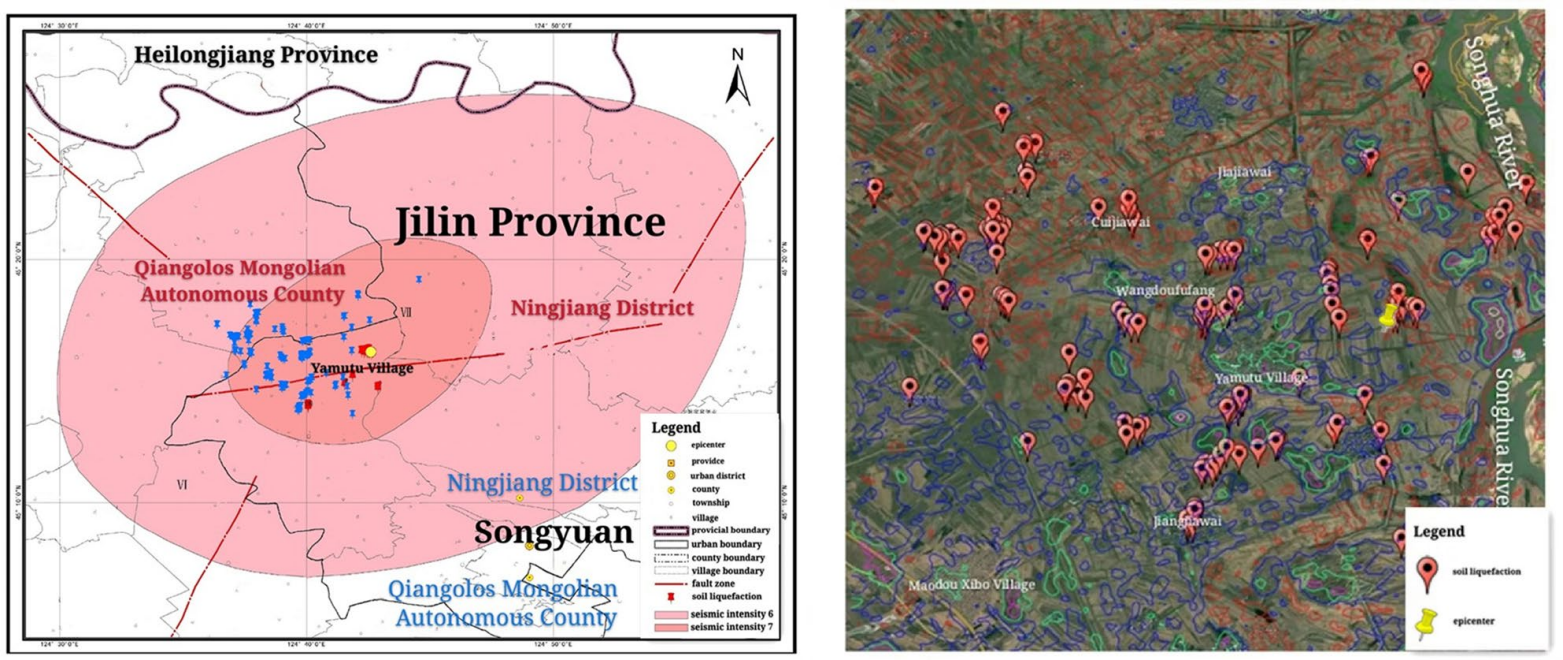
Distribution of liquefaction induced by Songyuan earthquake. We created this figure in using ArcGIS 10.0 (URL: http://www.esri.com/).

Field survey identified that large-scale liquefaction was observed at the region affected by the earthquake with a seismic intensity of VII. In addition, few liquefaction sites were found at the region which experienced the seismic intensity of VI. Figure 3 illustrates the distribution of the liquefied sites. All of the liquefied sites were located at the countryside, of which sand boil and water spout were the primary liquefaction macrophenomena. Majority of the liquefaction was identified on rice field and a small proportion of the liquefaction caused damage to buildings. Approximately two hundred liquefied sites were detected, most of which were distributed across the left bank of Songhua River. On the other hand, only one liquefied site was observed at the right bank. The liquefaction zone covered approximately 9 km in both width and length and (80 km^2^ area) with Yamutu Village at Ningjiang District as the center. The liquefaction zone spanned from Songhua river on the east to Langjia Village (Qianguo County) on the west, and Guojia Village (Qianguo County) on the north to Jiangjia Village (Ningjia District) on the south.

**Figure 4:**
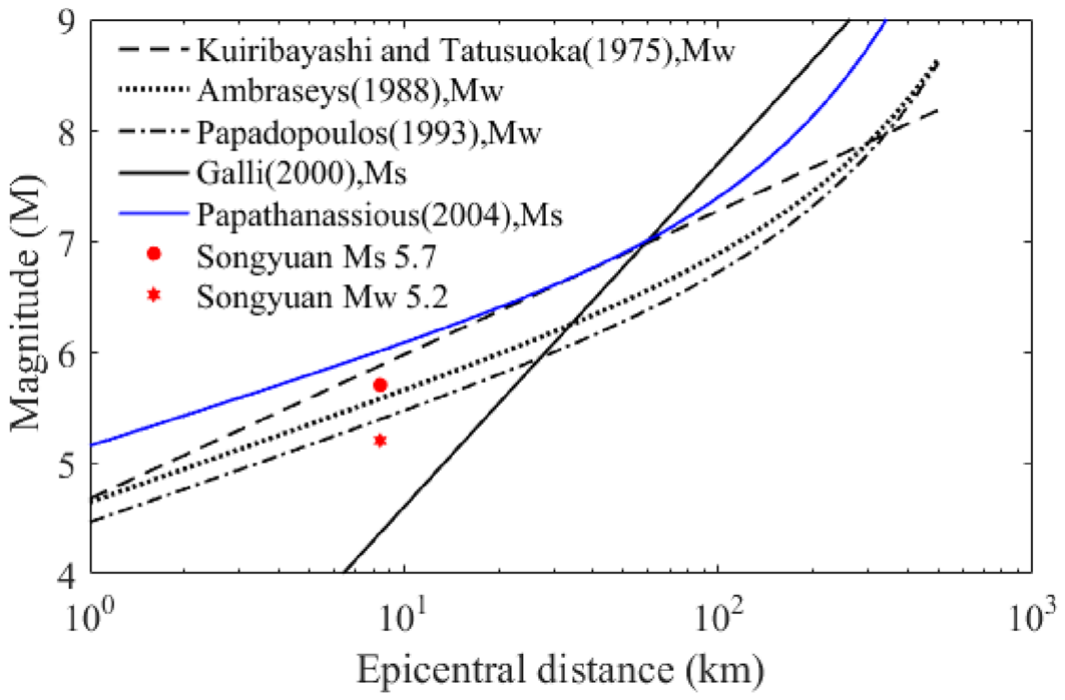
Comparison among the different curves about magnitude and maximum epicentral distance of liquefied sites provide by previous authors (The two dots represent the surface wave magnitude and the moment magnitude of this earthquake).

During this earthquake, maximum epicentral distance of the liquefied sites is 8.4 km. The relationship between the maximum epicentral distance of liquefied sites and earthquake magnitude (Mw or Ms) has been studied by many scholars (Kuiribayashi and Tatusuoka (1975), Ambraseys (1988), Papadopoulos and Lefkopoulos (1993), Galli (2000))^10,11^. Most liquefaction phenomena together with outcrop evidence have been observed in large earthquakes in terms of magnitude, but it is barely found in low magnitude earthquakes. A comparison among the different curves which show liquefaction occurrence in terms of earthquake magnitude provide by previous authors are presented in Fig. 4.

### Observation on liquefaction macrophenomena

**Figure 5:**
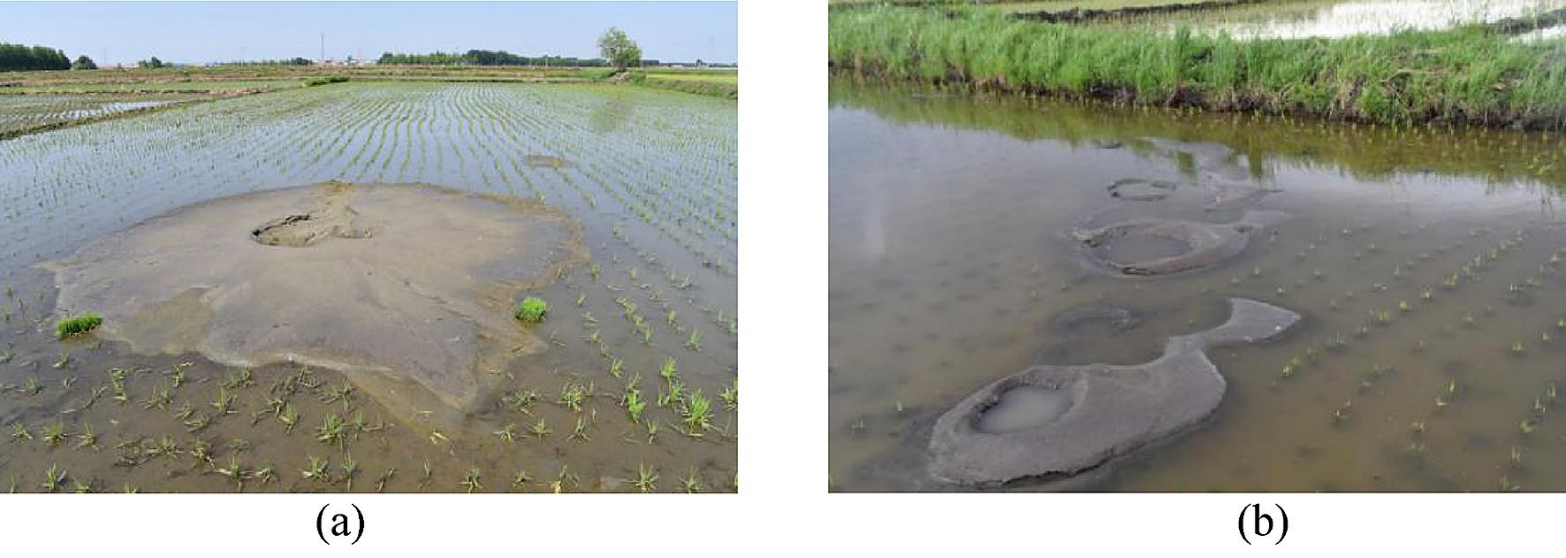
Typical macrophenomena observed on liquefaction sites (**a**) Concentric-shaped eruption with a direction of 28° and (**b**) string beads-shaped eruption at a direction of 354°.

Sand boil and waterspout were the main liquefaction macrophenomena discovered on liquefied sites. Majority of the macrophenomena was observed on rice field, and lesser proportion was found in forest, dry farmland and residential area. The liquefied sites discovered on rice field were the most severe among the discovered liquefied sites. The sites were characterized by long duration of liquefaction, deep liquefied hole, and large-scale eructation. Observed waterspout lasted from several minutes to several hours and irrigation water was still visible on a rice field at Jiangjia Village after four days of the earthquake, indicating that pressure of pore water in underground soil layer had not been fully dissipated Liquefied ejecta accumulated and were embodied as concentric (Fig. 5a) and string beads (Fig. 5b) shape. The diameter of most concentric shape was greater than 1 m and the largest recorded diameter was 10 m. The volume of the ejecta was approximately 10 m^3^. On the other hand, the string beads shape was embodied as a combination of multiple concentric shape, with the maximum length exceeded 20 m. The diameter of liquefied hole ranged from 20 cm to 4 m, with the maximum depth of 4 m.

**Figure 6:**
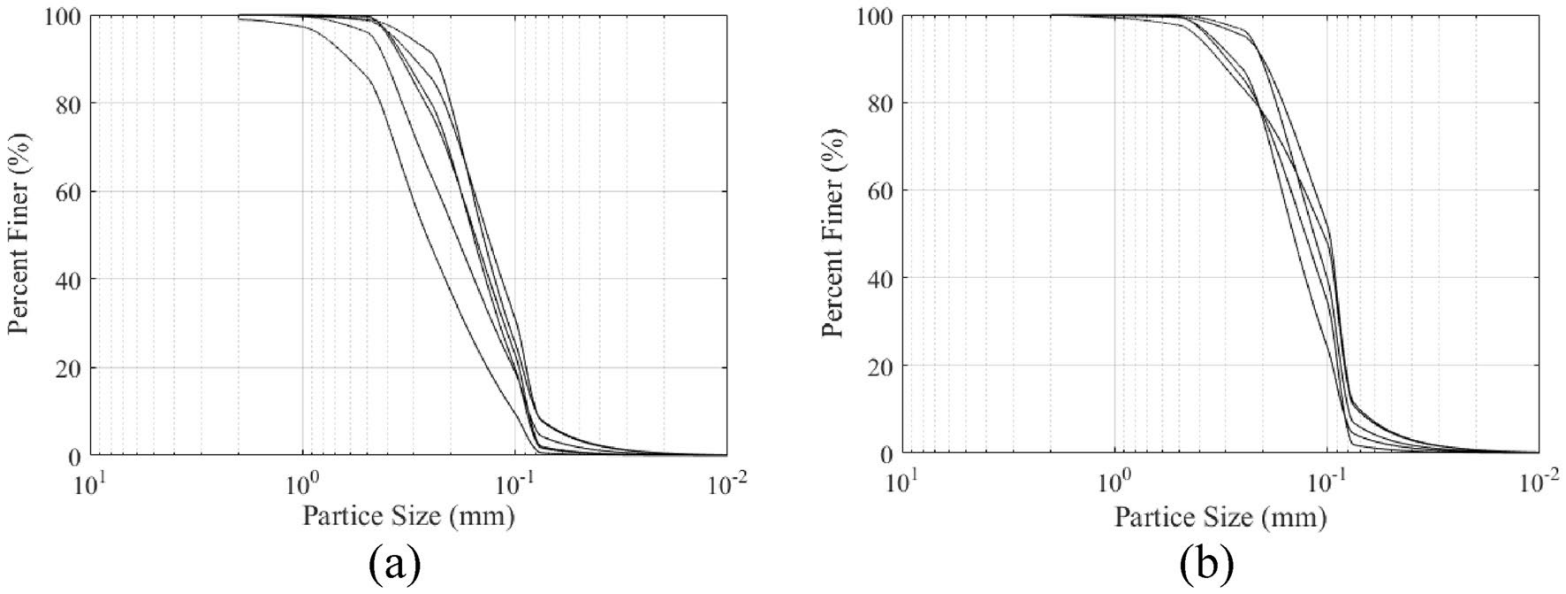
Particle size distribution of the two types liquefied sand (**a**) yellow fine sand (**b**) gray fine sand.

There are two main types of the liquefied sand ejected in this earthquake: yellow fine sand and gray fine sand. The yellow fine sand is more widely distributed than the gray fine sand, which mainly includes quartz and feldspar. The buried depth is about 3-7 m below the surface. The gray fine sand contains a certain amount of mica. The buried depth is generally greater than 5 m. The yellow fine sand is covered by the gray fine sand in part of the liquefied sites, this phenomenon is consistent with the buried relationship between the two types of sand. The particle size distribution of the two types liquefied sand are presented in Fig. 6. It is worth noting that samples are taken from the surface, not underground.

**Figure 7:**
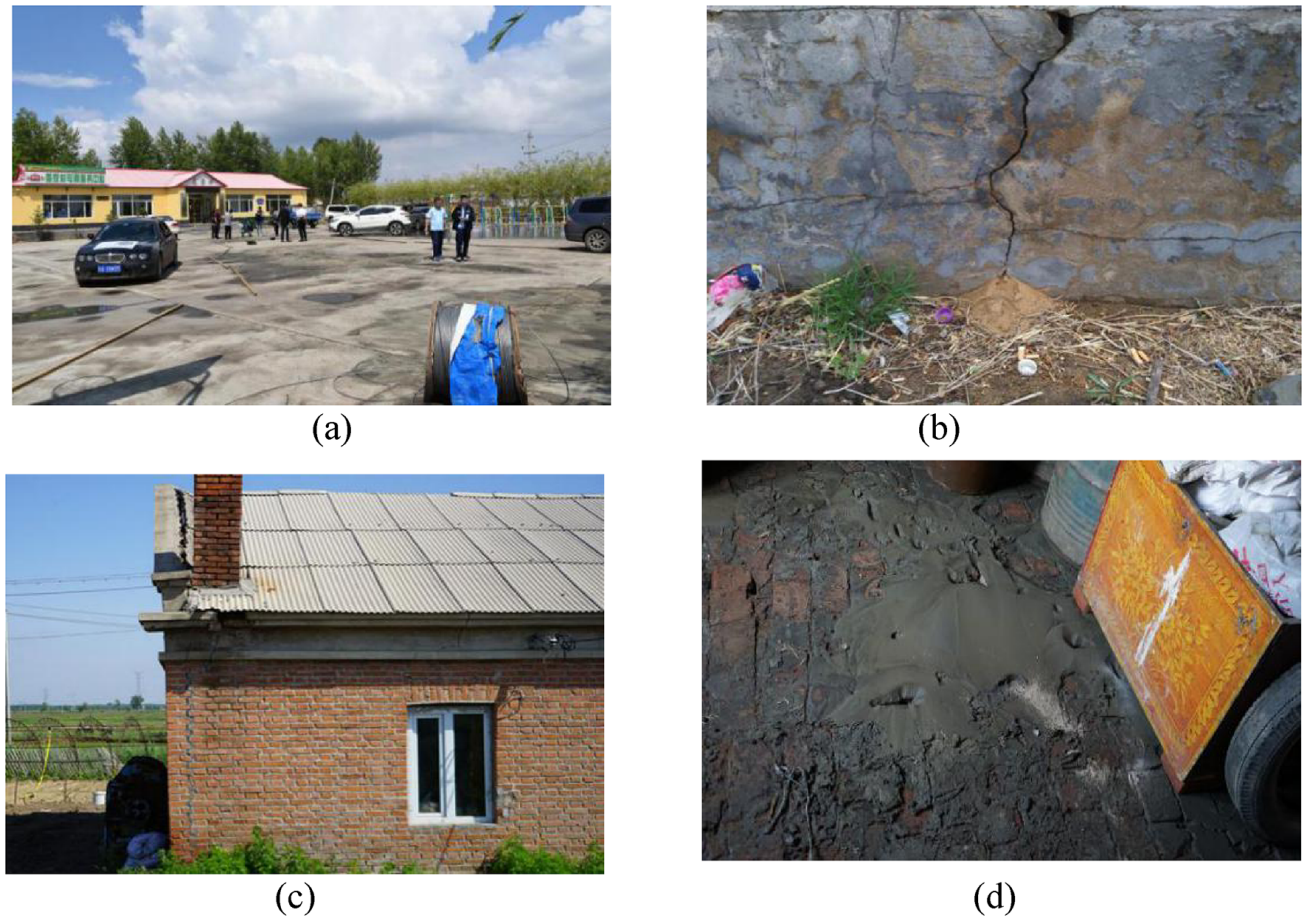
Construction damage due to liquefication: damage to a village committee office at Jiangjia (**a**,**b**) and damage to a house in Cuijia (**c**,**d**).

Despite the appearance of liquefaction macrophenomena, there was no extensive damage to building caused by the liquefaction. There were only two observed occasion on liquefaction-related damage to building: the damage to a village committee office at Jiangjia (Fig. 7a,b) and the damage to a house in Cuijia (Fig. 7c,d). These buildings are one-story masonry structures and liquefied sand (fine yellow sand and/or fine gray sand) were observed at both buildings Liquefied sand was exuded from the joint of 30 m × 40 m concrete floor of the village committee office at Jiangjia. Similarly, liquefied sand was expelled from the corner of the office’s foundation, despite producing only a slight damage to the building. The damage to the house in Cuijia was more pronounced than the damage to the village committee office at Jiangjia. The interior floor was fully covered by liquefied sand. Due to the absence of ring beam and construction column in the structure, liquefaction induced settlement and wall crack.

### Geotechnical property of earthquake sites

Based on the finding of field survey elaborated in "Distribution of liquefaction" and "Observation on liquefaction macrophenomena" section, drilling was carried out at the epicenter of the earthquake to the typical depth of soil to obtain data on soil layer as well as physical and mechanical index of the soil at liquefied and non-liquefied sites. Additionally, standard penetration test (SPT) and cone penetration test (CPT) were conducted to obtain valuable data for enriching previously reported liquefaction estimation method.

**Figure 8:**
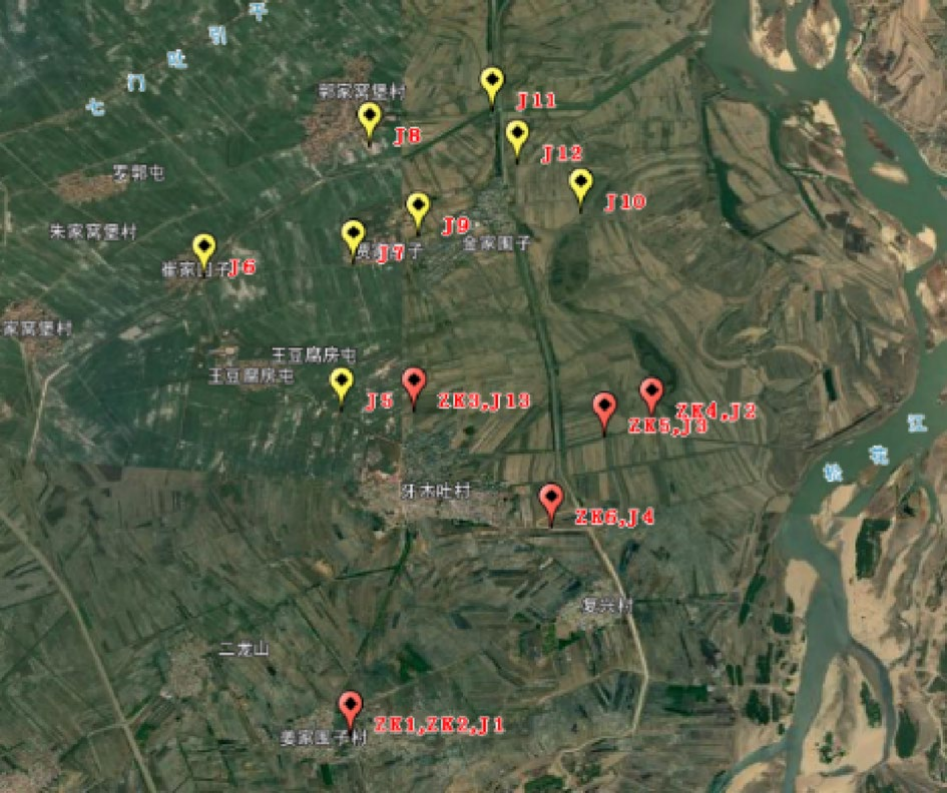
Sites at which drilling and cone penetration test were performed during field survey. ZK and J indicate the sites at which drilling and CPT were conducted respectively. We created this figure in using ArcGIS 10.0 (URL: http://www.esri.com/).

Figure 8 illustrates the sites at which drilling and CPT were performed. The sites were located at the region affected by the seismic intensity of VII. Drilling was performed at six sites, consisting of three sites at which sand eruption was identified and three sites at which sand eruption was not identified. Each drill hole reached the depth of approximately 20 m. Samples of soil layer were collected and subjected to standard penetration test on the other hand, CPT was performed at thirteen sites, of which sand eruption was identified at four of the sites. At six out of the thirteen sites, both drilling and CPT were carried out. The CPT employed a JMS-15A-3 probe with the cone bottom of 43.7 mm, cone tip area of 15 cm^2^, friction cylinder surface area of 300 cm^2^, and the cone angle of 60°. The weight of CPT truck was 20 ton. Researchers have been extensively investigating the application of CPT for studying liquefaction, and have obtained satisfactory result from field application^12–15^. Based on SPT and CPT data, liquefied soil layer was identified by the cone tip resistance and lateral friction resistance value collected at liquefied sites. While non-liquefied soil layer was characterized by high cone tip resistance and low lateral friction resistance, liquefied soil layer was identified by low cone tip resistance and low lateral friction resistance.

**Figure 9:**
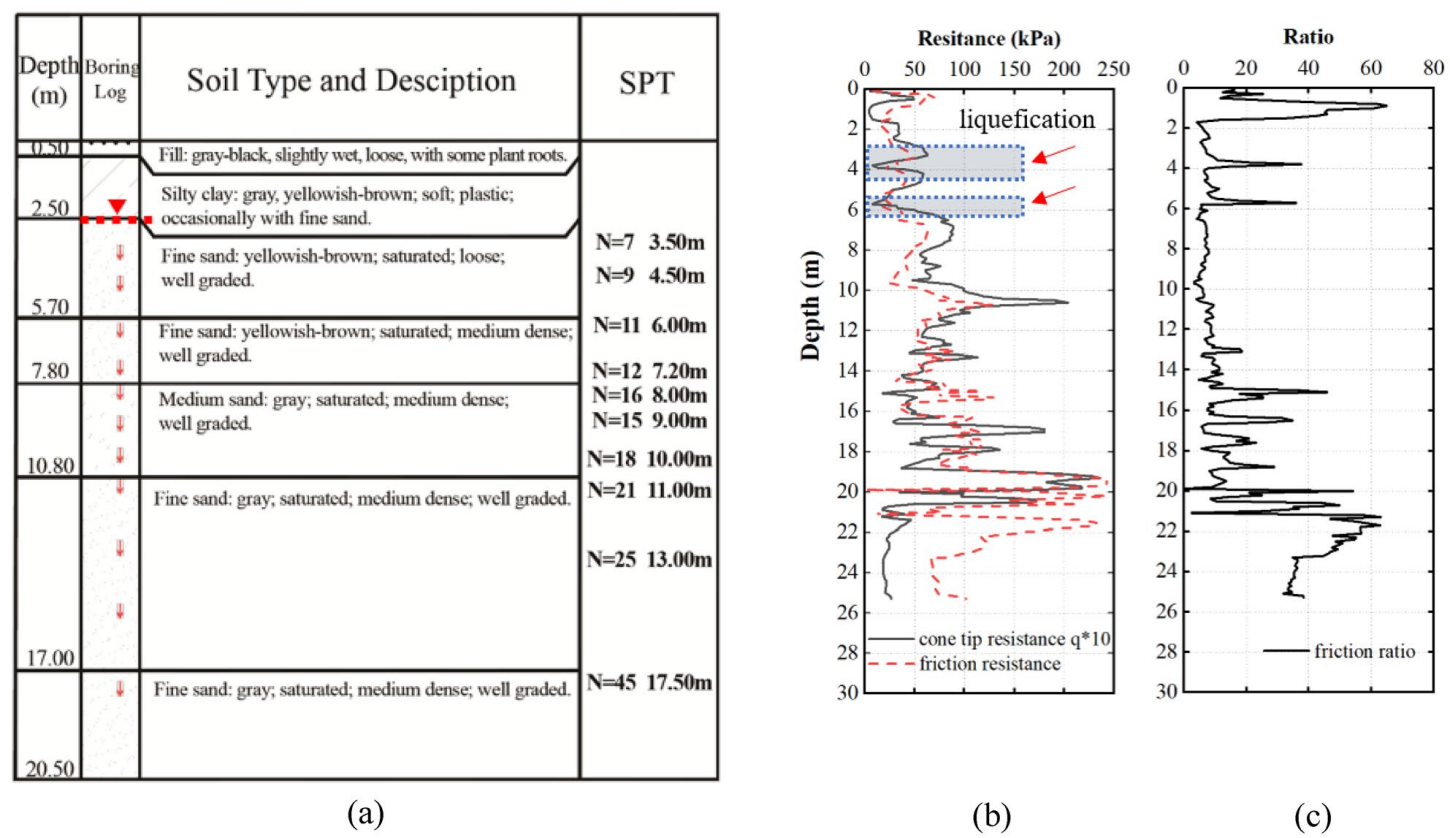
Soil profile (**a**), standard penetration test analysis (**b**), and cone penetration test analysis (**c**) of soil collected at liquefaction site.

Example of finding derived from the analysis of soil collected from a liquefied site, at which sand eruption was observed is presented in Fig. 9. The sampling site was located next to a rice field fully covered with water. Figure 9a indicates a simple profile of analyzed soil. The soil at the depth between 0 m and 2.5 m was composed of a layer of clay confining bed enriched with humus from plant root. Underground water was found at the 2.5 m depth. Successively, soil layer at the depth between 2.5 m and 20 m was filled with saturated fine yellowish-brown sand, medium gray sand, and fine gray sand successively. The soil layer at the depth greater than 20 m was constituted by a layer of clay confining bed. SPT and CPT data presented in Fig. 9b,c corresponded to the soil profile presented in Fig. 9a. Figure 9b,c show a clear distinction of each soil layer, as depicted by the value of cone tip resistance, lateral friction resistance, and friction ratio. More importantly, the SPT and CPT data clearly demonstrate the presence of a liquefied layer at the depth between 3 and 4 m. Overall, the finding presented in Fig. 9 supports the reliability of CPT to distinguish liquefied soil layer from the non-liquefied counterpart.

**Figure 10:**
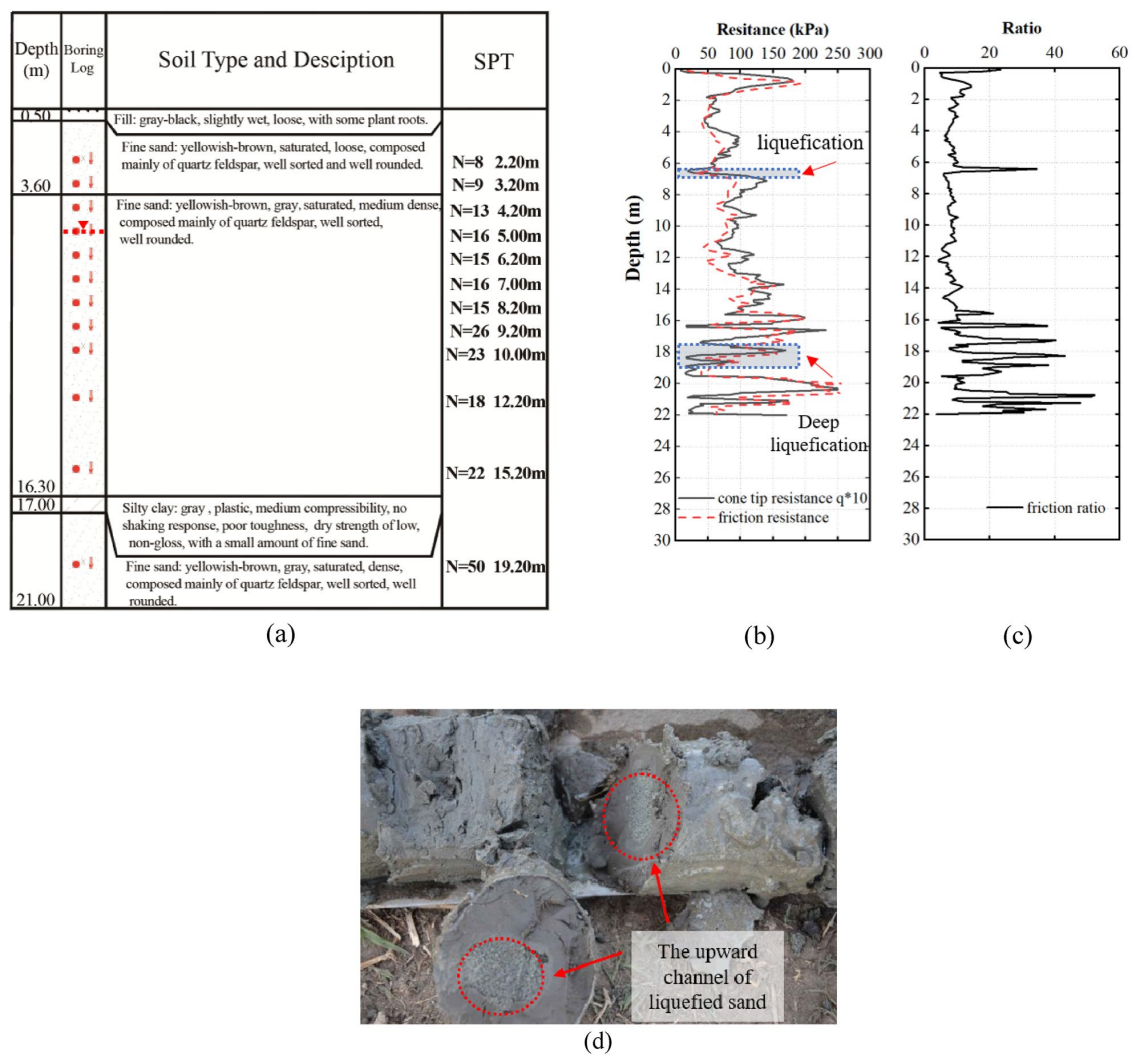
Soil profile (**a**), standard penetration test analysis (**b**), and cone penetration test analysis (**c**) of soil collected at non-liquefaction site. In addition, deep layer liquefaction (**d**) was observed at the site.

Figure 10 presents the example of finding derived from the analysis of soil collected from a non-liquefied site located next to a dry farmland. Underground water was discovered at a depth of 5 m, deeper than the depth of which underground water was discovered at the liquefied site. Nonetheless, the top layer of the soil had a similar composition to the top layer of soil collected from liquefied site: a clay layer enriched humus from plant root, which spanned from 0 m to 0.5 m depth. Successively, the soil layer was filled with yellow and gray sand of medium and/or high fineness until the depth of 17.0 m, at which grayish-black silty clay layer was detected. Underneath the silty clay layer, a layer of fine yellow and gray sand was detected. Visual inspection upon drilling demonstrated a slight liquefaction of the fine yellow sand layer at the 6–7 m depth, which agreed to the SPT and CPT data presented in Fig. 10b,c. More interestingly, deep liquefaction was observed. Liquefaction at lower sand layer generated an upward movement of liquefied sand towards the clay layer at the 17 m depth, as visually demonstrated by the drilled sample (Fig. 10d). The occurrence of the deep sand liquefaction was further ascertained by the low value of cone tip resistance and lateral friction resistance at the depth between 18 and 19 m (Fig. 10b).

Seed and Idriss^16,17^ proposed a simplified calculation formula of sand liquefaction cycle stress ratio:

1 $$ CSR = \frac{{\tau_{av} }}{{\sigma_{v} }} = 0.65\frac{{\alpha_{\max } }}{g}\frac{{\sigma_{v} }}{{\sigma_{v}^{\prime } }}r_{d} MSF^{ - 1} $$

where, CSR is cyclic stress ratio, $a_{\max }$ is peak horizontal acceleration, g is acceleration of gravity, $\sigma_{v}$ is vertical total stress, $\sigma_{{\text{v}}}^{^{\prime}}$ is vertical effective stress, $\gamma_{d}$ is stress reduction coefficient, MSF is magnitude scale factor.

CRR is cyclic resistance ratio. When CSR > CRR, the soil layer is judged to be liquefied; otherwise, it is judged to be non-liquefied. Rauch (1996) proposed a formula to calculate CRR:

2 $$ CRR_{7.5} = \frac{1}{{300 - (N_{{1}} )_{{60{\text{cs}}}} }} + \frac{{(N_{{1}} )_{60cs} }}{135} + \frac{50}{{[10(N_{{1}} )_{60cs} + 45]^{2} }} - \frac{1}{200} $$

where, N_1,60cs_ is fines-corrected standard penetration test blow count corrected for overburden,energy and procedural differences.

**Figure 11:**
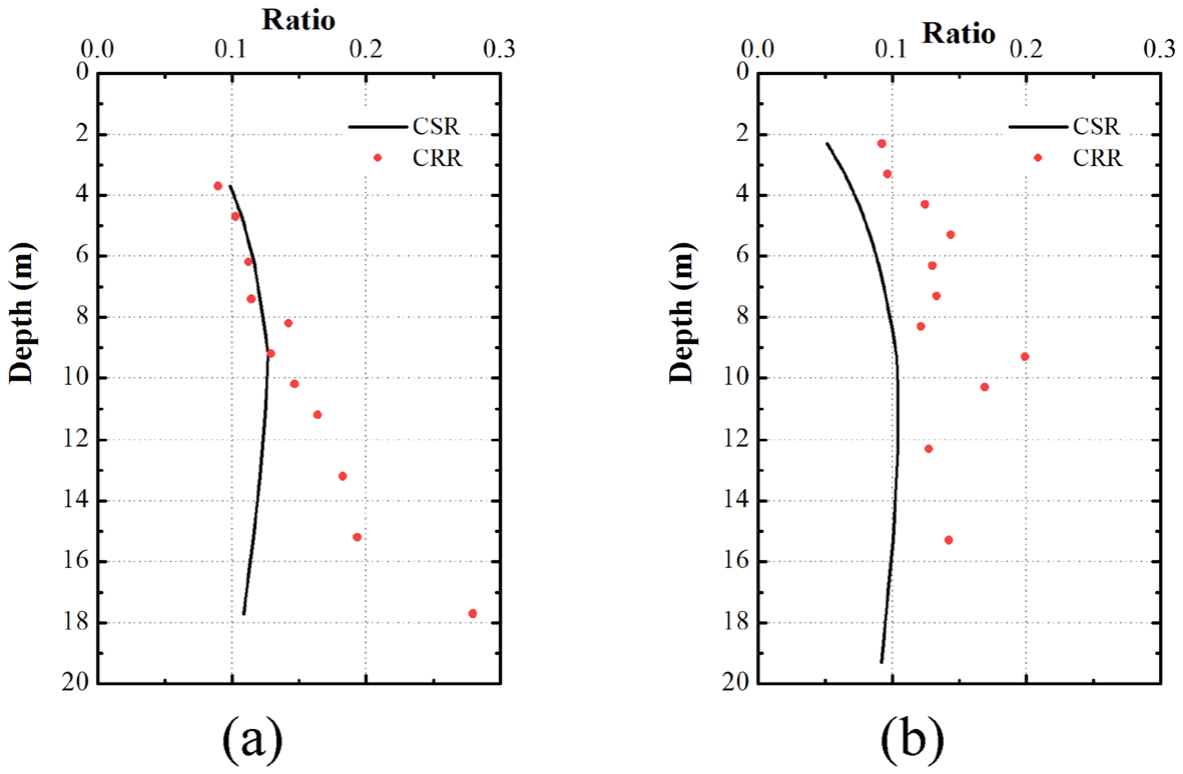
Seed-Idriss method’s point at (**a**) liquefaction and (**b**) non-liquefaction site.

Seed-Idriss method’s point at liquefaction and non-liquefaction site are presented in Fig. 11.This method can gives an accurate prediction to the Shallow sand liquefaction (Fig. 11a).For deep liquefaction under 17 m, this method gives a error prediction (Fig. 11b).

## Macrophenomena of loess liquefaction

### Basic appearance

**Figure 12:**
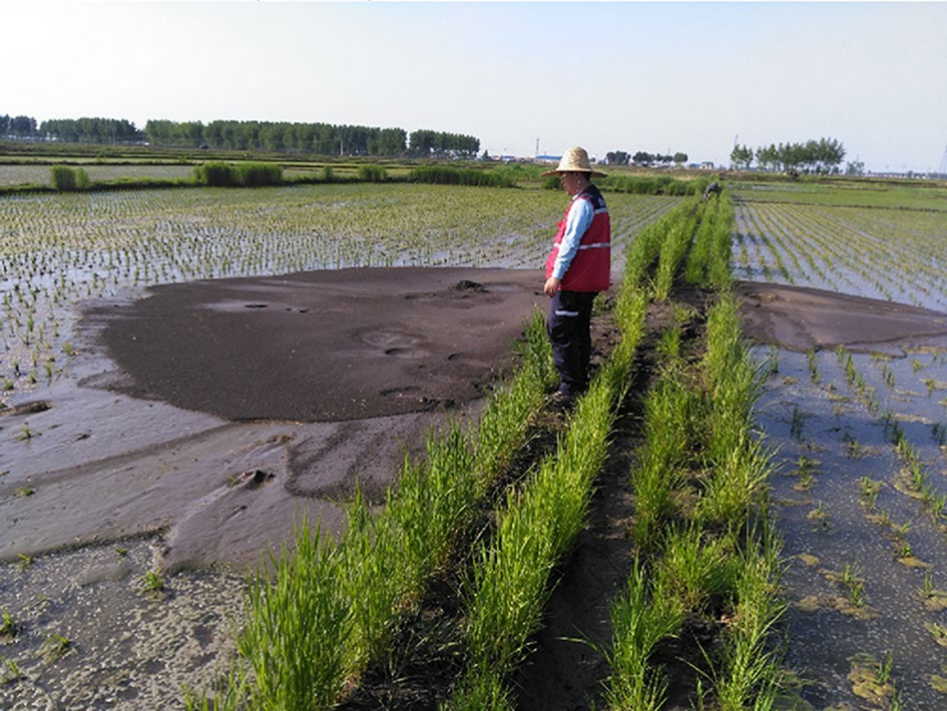
Special soil liquefaction discovered on a rice field at Wangjia Village. The participant included in the study has provided informed consent to publish the image.

Special soil liquefaction was located at Wangjia Village (Fig. 12). The liquefied soil resembled silty clay and was constituted of grayish-black particle. The diameter of the ejecta was approximately 6 m and the thickness was approximately 15 cm. The soil was soft yet viscous, and rollable into clay strip. In order to determine the type of the liquefied soil, micromorphology and chemical composition of the soil were analyzed by scanning electron microscopy (SEM), X-ray diffraction (XRD), and X-ray fluorescence (XRF).

**Figure 13:**
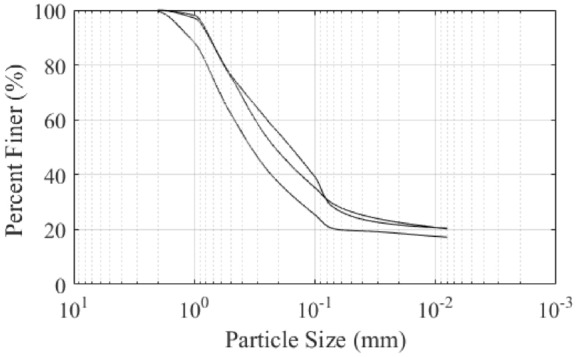
Particle size distribution of the special soil.

The soil sample is black grain when saturated with water. After drying, it is earthy yellow and can see some particles.The Liquid-plastic limit test and particle size analysis result are presented in Table 1 and Fig. 13. According to the test results, the special soil is a type of clay.

**Table 1:** The Liquid-plastic limit test and particle size analysis result of the special soil.

Plastic limit (%)	Liquid limit (%)	Plasticity index	Clay content (< 0.075 mm) (%)
33	46	26	20.5

### Micromorphology and composition analysis

**Figure 14:**
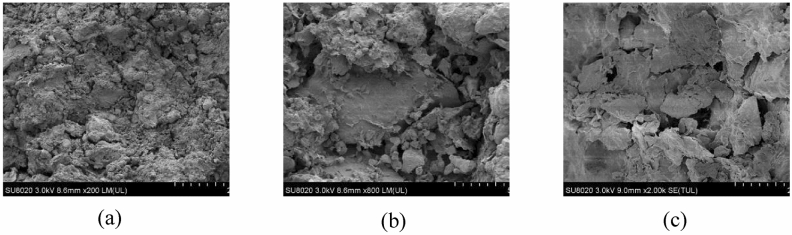
Scanning electron microscope image of liquefied soil at increasing magnification: (**a**) 200×, (**b**) 800×, and (**c**) 2000×.

**Figure 15:**
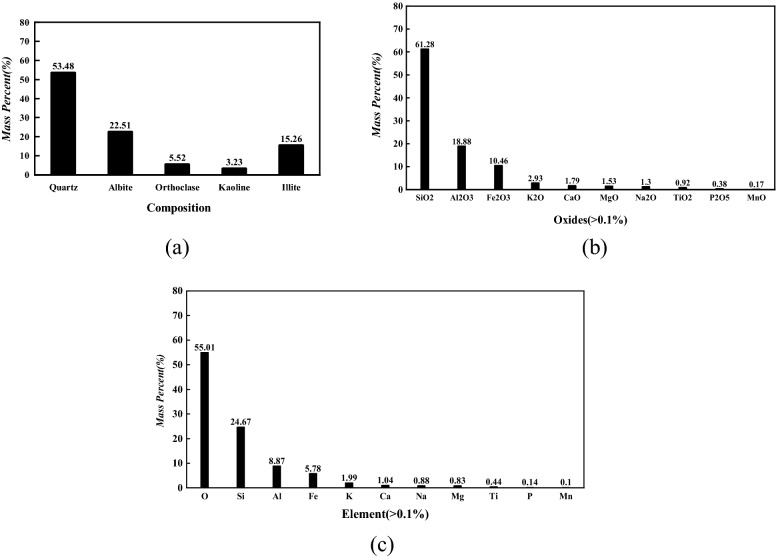
Mineral (**a**), elemental oxide (**b**), and element (**c**) composition of liquefied soil.

Micromorphological characterization of rare liquefied soil collected from ground surface was performed with Hitachi SU8020 field emission scanning electron microscope (accelerating voltage = 3 kV), and is presented in Fig. 14. As suggested in Fig. 14, the liquefied soil was composed of not only round sand particle but also flat clay particle, with the flat clay particle being more abundant than the round particle counterpart. Moreover, the primary structure of the soil was damaged, and small and medium pores constituted a greater proportion than did the large pore. Furthermore, the particle was contacted by (basal or contact) cementation. Based on the visual appearance (i.e. resemblance to silty clay) and elaborated micromorphological characteristic of the liquefied soil, it was postulated that the sample was liquefied loess. The hypothesis was supported by Bo et al. who reported the distribution of sub-clay or loess layer in the investigated area, which was located at a shallow depth below the ground. Moreover, previous field and laboratory studies have reported the liquefaction of saturated loess under earthquake with the intensity of VI. The identity of liquefied soil was further verified through chemical composition analysis by XRD and XRF, presented in Fig. 15.

As demonstrated in Fig. 15, quartz was the major mineral of the liquefied soil (53.48%), followed by albite (22.51%), illite (15.26%), orthoclase (5.52%), and kaoline (3.23%). On the other hand, SiO_2_ and Al_2_O_3_ were the most abundant elemental oxide, and oxygen was the major element of the soil. The major content of quartz in the liquefied soil was in agreement with the report by Wei that quartz was a major mineral of Chinese loess^18^. Thus, the mineral composition of liquefied soil was compared with the mineral composition of Malan loess reported by Liu et al. and is presented in Table 2. The SiO_2_ content of liquefied soil conformed with the reported SiO_2_ content of Malan loess, so did the Al_2_O_3_ content. On the other hand, the discrepancy in Fe_2_O_3_ and CaO content was justified by the sampling limitation: the liquefied soil was collected above the ground, thus exposing the sample to oxygen from atmosphere. The exposure induced the oxidation of Fe^2+^ to Fe^3+^ thereby producing a higher Fe_2_O_3_ content. Meanwhile, carbonate was dissolved, which reduced the CaO content. In addition to the similarity in mineral composition, the similarity in physical appearance (resemblance to silty clay) affirmed that the liquefied soil was liquefied loess, as further reflected by the comparable physical property (Table 2).

**Table 2:** Mineral composition and physical property of liquefied soil and Malan loess.

Soil sample	Mineral composition (%)				Physical property	
	SiO_2_	Al_2_O_3_	Fe_2_O_3_	CaO	Plasticity index	Clay content (< 0.075 mm)(%)
Liquefied soil	61.3	18.9	10.5	1.8	13	20.5
Malan loess (adapted from Liu.)	> 60		3 ~ 6	7.5 ~ 10.5	7.6 ~ 15.7	7.0 ~ 30.4

Overall, based on visual inspection, micromorphology analysis, chemical composition analysis, and physical property comparison, the authors postulated that the observed rare liquefied soil was liquefied loess. It is important to emphasize that there has been no study reporting the discovery of liquefied loess in Northeast China. Unfortunately, due to time and financial constraint, neither drilling was performed at the site nor undisturbed soil sample was collected. Hence, further investigation is required to study the formation mechanism, factors governing the formation, and physical property of the postulated liquefied loess (e.g. natural density and moisture content).

## Conclusion

Based on conducted post-earthquake field survey and subsequent analytical procedure (soil profile determination, CPT and SPT analysis, micromorphological study by SEM, and chemical composition analysis by XRD and XRF) at the epicenter of M5.7 Songyuan earthquake, the following conclusion was drawn:

1. Liquefaction was observed at the epicenter, and caused surface damage mainly to rice field. Nonetheless, there was no large-scale damage to building. Due to the simple profile of regional soil layer, the distribution of liquefaction was primarily governed by ground motion, landform, and groundwater level.
2. Deep liquefaction. Most liquefaction was identified at shallow depth: at soil layer with the depth less than 10 m. The liquefaction produced surface damage and was easily discernable by the phenomena such as sand boil and water spout. On the other hand, deep liquefaction is commonly neglected in field investigation. Nonetheless, in this paper the authors reported the upward movement of liquefied sand from the lower sand layer towards the upper clay layer located at the depth between 16 and 17 m, suggesting the occurrence of deep liquefaction at the depth of between 18 and 19 m. This phenomenon is rarely reported by other field investigation. Additionally, the accuracy of existing method to determine the occurrence of deep liquefaction is low, hence requiring further improvement.
3. In-situ determination method of liquefaction. This study reported the occurrence of liquefaction at the sites with no apparent surface macrophenomena (e.g. sand boil and water spout). Unfortunately, existing field investigation method (drilling and SPT) are not practical for identifying such liquefaction occurrence. Hence, alternative method which is more efficient and accurate is necessitated. CPT is a candidate for the alternative method. It requires less time and produces more accurate result than the current drilling and SPT method. Moreover, it is applicable in-situ during post-earthquake investigation for identifying sand liquefaction. Nevertheless, given the recent incorporation of the method into in-situ post-earthquake investigation, more field data are required to improve the reliability of the method.
4. Special soil liquefaction. In addition to the typical sand liquefaction, special soil liquefaction, specifically loess liquefaction, was postulated. This study is the first study which reported the occurrence of loess liquefaction at Northeast China. Nonetheless, further investigation is required to understand the formation mechanism and factors controlling the formation and/or distribution of the loess liquefaction.
5. High risk of liquefaction at Songyuan. Songyuan is one of the only two cities in Northeast China which is fortified against seismic intensity of VIII. The local seismic activity is frequent and characterized by multiple earthquake swarm. The soil layer is constituted by thick loose sand and the groundwater is located at a shallow depth. Overall finding of this study suggests a high risk of liquefaction at Songyuan, contributed by not only typical sand liquefaction but also special soil liquefaction such as the postulated loess liquefaction. Therefore, research dedicated to studying the liquefaction at Songyuan is essential for improving planning on earthquake mitigation and design project.
